# Effectiveness of a Self-monitoring Device for Urinary Sodium-to-Potassium Ratio on Dietary Improvement in Free-Living Adults: a Randomized Controlled Trial

**DOI:** 10.2188/jea.JE20160144

**Published:** 2018-01-05

**Authors:** Toshiyuki Iwahori, Hirotsugu Ueshima, Naoto Ohgami, Hideyuki Yamashita, Naoko Miyagawa, Keiko Kondo, Sayuki Torii, Katsushi Yoshita, Toshikazu Shiga, Takayoshi Ohkubo, Hisatomi Arima, Katsuyuki Miura

**Affiliations:** 1 OMRON Healthcare Co., Ltd., Kyoto, Japan; 2 Department of Public Health, Shiga University of Medical Science, Shiga, Japan; 3 Center for Epidemiologic Research in Asia, Shiga University of Medical Science, Shiga, Japan; 4 Department of Hygiene and Public Health, Teikyo University of Medicine, Tokyo, Japan; 5 Department of Food and Human Health Science, Osaka City University, Osaka, Japan; 6 Department of Preventive Medicine and Public Health, Fukuoka University, Fukuoka, Japan

**Keywords:** lifestyle modification, potassium, self-monitoring, sodium, sodium-to-potassium ratio

## Abstract

**Background:**

Reducing the urinary sodium-to-potassium ratio is important for reducing both blood pressure and risk of cardiovascular disease. Among free-living Japanese individuals, we carried out a randomized trial to clarify the effect of lifestyle modification for lowering urinary sodium-to-potassium ratio using a self-monitoring device.

**Methods:**

This was an open, prospective, parallel randomized, controlled trial. Ninety-two individuals were recruited from Japanese volunteers. Participants were randomly allocated into intervention and control groups. A month-long dietary intervention on self-monitoring urinary sodium-to-potassium ratio was carried out using monitors (HEU-001F, OMRON Healthcare Co., Ltd., Kyoto, Japan). All participants had brief dietary education and received a leaflet as usual care. Monitors were handed out to the intervention group, but not to the control group. The intervention group was asked to measure at least one spot urine sodium-to-potassium ratio daily, and advised to lower their sodium-to-potassium ratio toward the target of less than 1. Outcomes included changes in 24-hour urinary sodium-to-potassium ratio, sodium excretion, potassium excretion, blood pressure, and body weight in both groups.

**Results:**

Mean measurement frequency of monitoring was 2.8 times/day during the intervention. Changes in urinary sodium-to-potassium ratio were −0.55 in the intervention group and −0.06 in the control group (*P* = 0.088); respective sodium excretion changes were −18.5 mmol/24 hours and −8.7 mmol/24 hours (*P* = 0.528); and corresponding potassium excretion was 2.6 mmol/24 hours and −1.5 mmol/24 hours (*P* = 0.300). No significant reductions were observed in either blood pressure or body weight after the intervention.

**Conclusions:**

Providing the device to self-monitor a sodium-to-potassium ratio did not achieve the targeted reduction of the ratio in “pure self-management” settings, indicating further needs to study an effective method to enhance the synergetic effect of dietary programs and self-monitoring practice to achieve the reduction. However, we cannot deny the possibility of reducing sodium-to-potassium ratio using a self-monitoring device.

## INTRODUCTION

High dietary sodium (Na) and low dietary potassium (K) intakes are associated with adverse blood pressure (BP) levels and excess risk of cardiovascular disease (CVD).^1^^–^^6^ Epidemiological studies also demonstrated that there was a good correlation between Na/K ratios in 24-hour urine and BP.^5^^–^^13^ The Na/K ratio has also been reported to be a superior metric to either Na or K alone in relation to BP and incident hypertension^5^^–^^7^^,^^14^^,^^15^; studies also report associations between Na/K ratio and CVD.^16^^–^^18^ The World Health Organization (WHO) plans to reduce Na intake and increase K intake in the future.^19^^,^^20^ However, a conventional population approach does not seem sufficient^21^; fairly large gaps exist between the actual intakes and the recommended target levels among populations.^5^^,^^22^^,^^23^ An individual approach, such as self-monitoring of Na and K intake, is also necessary for the timely achievement of goals set by the WHO — less than 2 g/day of Na by 2025.^24^ However, awareness and the actual practice toward individual intake goals remain poor; individuals who reported intakes while presumably consuming reduced and non-reduced salt diets showed similar levels of Na intake.^25^

The gold standard for estimating individual daily salt intake is 24-hour urine collection.^26^^–^^28^ The amount of K excreted in 24-hour urine does not necessarily reflect absolute K intake, not as well as it does for Na, but it shows a good correlation with dietary K intake.^27^^–^^29^ However, 24-hour urine collection is neither easy nor practical for patients at clinics or in their homes. There is little evidence for dietary intervention to reduce Na and increase K based on a behavioral theoretical framework. Effective monitoring of adherence to the recommended dietary Na and K intake in hypertensive patients and general populations requires development of a convenient, cheap, and appropriate monitoring system. Thus, a new self-monitoring device for supporting Na reduction and K increase is needed. Nowadays, self-monitoring devices for urinary Na/K ratio gives individuals quick feedback of their urinary Na/K ratio.^30^ However, the intervention effect of this self-monitoring device in supporting dietary behavior modification and changes in urinary Na/K ratio has not yet been thoroughly investigated. Thus, this randomized controlled trial was carried out to clarify the effects of lifestyle modification using a urinary Na/K ratio monitoring device among free-living Japanese individuals.

## METHODS

### Study design

This study is an open, prospective, parallel randomized controlled trial. This intervention trial was registered at the Japanese University hospital Medical Information Network Clinical Trials Registry (UMIN-CTR) as UMIN000007241.

### Participants

**Figure 1.**
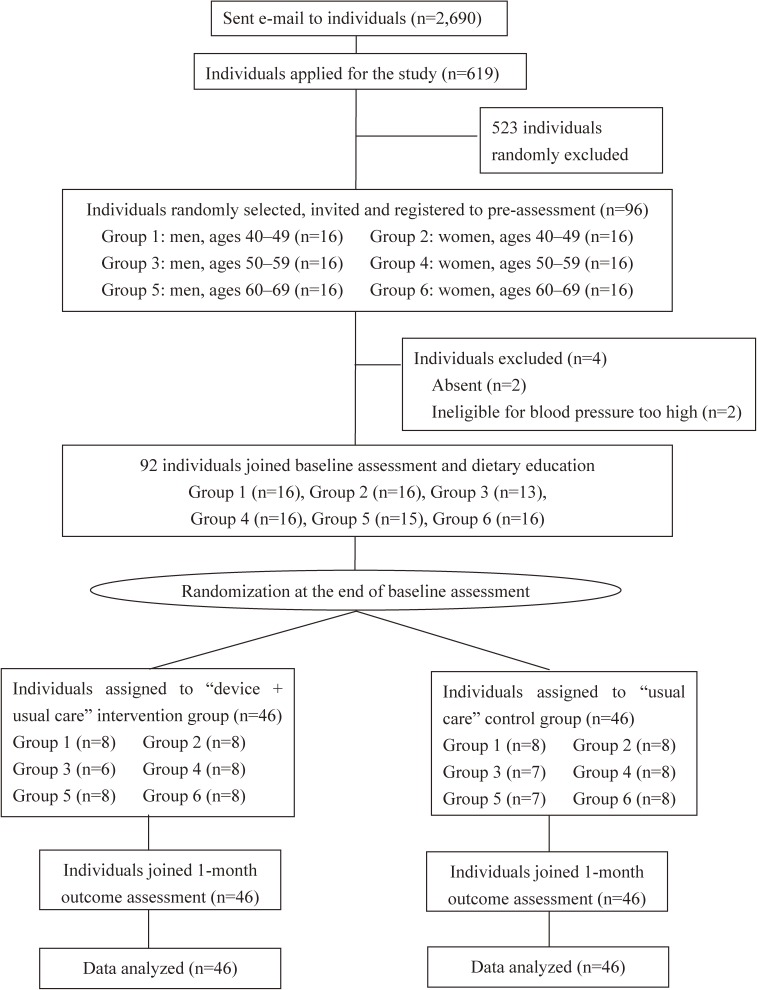
Participant flow in this randomized trial. Participant flow, starting from the initial application until the time of the 1-month outcome assessment, of this intervention trial. Participants initially received an email asking if they would like to take part in the intervention trial. After volunteering, the participants were first registered and then participated in this randomized trial. The intervention lasted for approximately a month.

Subjects were recruited via email from December 2011 through January 2012. We sent emails to healthy or hypertensive individuals in Kyoto, Japan and the surrounding areas, and the study was conducted from January 2012 through March 2012. An identification number was provided to participants during the registration period. Menstruating women; individuals with secondary hypertension, diabetes, or chronic kidney disease (CKD); individuals with a history of diabetes, CVD, cerebrovascular disease, or CKD; and participants who did not undergo baseline assessment or who met the high BP exclusion criteria (systolic BP above 180 mm Hg, diastolic BP above 110 mm Hg) during the initial baseline exam were not included in this study. Participants were placed into the appropriate 10-year age-gender group, with approximately equal numbers of participants registering in each group (Figure 1 and eFigure 1). All eligible participants were invited to a randomization session, where a computer-generated numbered sequence was prepared by an investigator in order to conduct appropriate randomization for this trial. Thus, eligible study participants were randomly allocated into two groups using a stratified random sampling procedure based on a computer-generated numbered sequence. The result of the allocation was communicated to the participant by an investigator who was blinded to participant’s information. Written informed consent was obtained from all participants. The ethics committees of OMRON Healthcare Co., Ltd. and Shiga University of Medical Science approved this study.

### Sample size

At baseline, we assumed the urinary Na/K ratio as 4.3; Na excretion as 188.4 mmol (11.0 g of salt); and K excretion as 45.5 mmol (1.8 g).^31^^–^^35^ Since this is the first trial on the effects of self-management on urinary Na/K ratio using a self-monitoring device, we were unable to reference the findings of previous intervention studies. Thus, we referred to findings from observational studies and assumed that the intervention effect would induce a level close to the average value of either worldwide populations (urinary Na/K ratio of 3.3) or Western populations (urinary Na/K ratio of 2.2), which had lower Na/K ratios than we initially expected.^31^^–^^34^ If we assume that the reduction in urinary Na/K ratio is 2.1 (SD 1.5) in the intervention group and 1.0 (SD 1.5) in the control group (pre-specified effect size: 1 unit difference in Na/K ratio among these two groups), then a sample size of 36 participants in each group would be needed to detect a significant difference occurring with a two-sided alpha = 0.05 and beta = 0.20. Therefore, the power to detect the difference (1 − beta) was 80% in this study. Considering losses to follow-up, a sample size of 48 for each group were invited and registered in this study (Figure 1).

### Interventions

**Figure 2.**
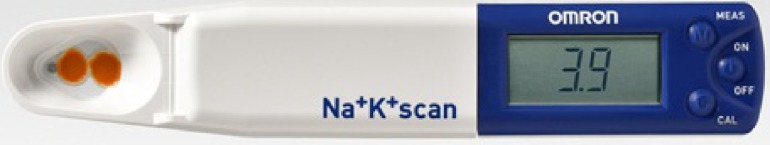
Self-monitoring device used in the randomized trial. Portable handy-sized urinary Na/K ratio monitor (HEU-001F, OMRON Healthcare Co., Ltd., Kyoto, Japan) used for self-monitoring of the dietary lifestyle of the participants during the randomized trial. This device measures urinary Na and K concentrations, and calculates urinary Na/K ratio via ion selective electrodes. Only participants in the “device + usual care” intervention group were given the monitoring device. These participants were then asked to measure at least one spot urine Na/K ratio daily.

Eligible study participants were randomly allocated into two groups. All subjects in the “device + usual care” intervention group, but not those in the “usual care” control group, were given portable handy-sized urinary Na/K ratio monitors (HEU-001F; OMRON Healthcare Co., Ltd., Kyoto, Japan; Figure 2, eFigure 2, and Supplemental Video). Participants in the intervention group were asked to measure at least one spot urine Na/K ratio daily, and to try to reduce their Na and increase their K intake in order to lower their urinary Na/K ratio to achieve a target set by investigators. Prior to the intervention, all participants received a brief dietary education that provided details on how to avoid high salt foods and which vegetables and fruits could be consumed in order to increase K intake. For salt reduction, participants were given information on the top 10 Na intake sources in Japanese meals and were then advised on how to avoid these foods by replacing them with other reduced-Na alternatives.^36^ To increase the K, participants were provided with information on the major K intake sources that were available in local grocery stores. After this educational session, every participant received a leaflet that summarized this dietary information prior to the start of the 1-month intervention trial. After the intervention, the 1-month outcome was assessed in each of the participants (Figure 1).

### Target levels of Na/K ratio during the self-monitoring intervention

Although there has yet to be any definitive guidelines for recommended levels of urinary Na/K ratio, the levels of the mean urinary Na/K ratio in many countries around the world remains above 3.00.^5^^,^^22^^,^^31^^–^^35^ A recent report from the TOHP follow-up study has reported a lower level of cardiovascular risk with urinary Na/K ratio less than 2.00.^16^ We advised all participants to target a urinary Na/K ratio of less than 1.00, since WHO has suggested that achieving recommended levels of both Na and K would deliver a Na/K ratio of approximately 1.00.^19^^,^^20^ Also, findings from the INTERSALT study suggest that adjusting an individual’s lifestyle to achieve a Na/K ratio of less than 1.00 would be beneficial for health.^6^

### Baseline and 1-month outcome assessment

Participants in both groups completed 24-hour urine collection and underwent measurements for BP, body weight, and height during the baseline and 1-month outcome assessments (Figure 1). Participants were instructed to collect all urine samples using a measuring cup, with all urine saved in 1 L bottles, unless urine collection was unsuccessful or contaminated by feces. The 24-hour urine collection began with the participant voiding their urine during their first visit, and finished during the visit on the following day when the participant returned with their 24-hour urine collection bottles. Urine samples were transferred into an aliquot tube, with all the samples then sent to the biochemical inspection center (BML Inc., Tokyo, Japan).

BP, weight, and height were measured at the time just after the start and the end of the 24-hour urine collection. BP was measured by certified technicians using an automated BP monitor (HEM-7081IT; OMRON Healthcare Co., Ltd., Kyoto, Japan). After the participant sat quietly for 5 minutes, BP was measured twice in the right arm with an appropriate size cuff at an ambient temperature that was above 20 degrees centigrade. At each assessment point, the mean of four measurements was used for BP. Weight was measured twice using a body composition monitor (HBF-206IT; OMRON Healthcare Co., Ltd.), while the height was measured using a stadiometer (HP-M; Tsutsumi, Tokyo, Japan); both measurements were performed in all patients without shoes.

### Outcomes

The primary outcome used in this study was the change in 24-hour urinary Na/K ratio. Secondary outcomes included changes in the 24-hour excretion of Na and K, BP, and body weight.

### Analysis

The measured data were entered into the computer by two workers and merged together as an overall dataset. The investigator then checked the distribution of the variables before the analyses; data were not skewed, and no outlier was evident. The reduction in the Na/K ratio and the other outcomes between the intervention group and the control group were compared using an unpaired t-test procedure in SAS version 9.4 (SAS Institute, Cary, NC, USA). Statistical significance was defined as a two-tailed *P*-value of <0.05. The evaluation method was pre-specified in the protocol and approved by ethics committees of Shiga University of Medical Science and OMRON Healthcare Co., Ltd.

## RESULTS

As shown in Figure 1, out of 2,690 emails sent to healthy or hypertensive individuals who had participated in our previous study of BP measurement, 619 individuals responded and volunteered to participate in this trial. A total of 96 individuals (48 men and 48 women, ages 40 to 69 years) were randomly selected from the pool of applicants. We performed the study from January through March, 2012, and a total of four participants were excluded during the pre-assessment: two individuals due to absence during the baseline assessment, and two other individuals who met the high BP exclusion criteria (systolic BP above 180 mm Hg, diastolic BP above 110 mm Hg). A total of 92 eligible individuals (44 men and 48 women, ages 40 to 69 years, with a mean age of 55 years) were randomly allocated into two groups.

### Baseline characteristics

Table 1 shows a comparison of the baseline data for both groups. Out of the total group of participants, 22% were hypertensive individuals (*n* = 20), with 16% of these individuals on anti-hypertensive medication (*n* = 15). The anti-hypertensive medications administered to 5 (33%), 3 (20%), 2 (13%), 1 (7%), 1 (7%), and 3 (17%) participants were calcium channel blockers (CCB), angiotensin 2 receptor blockers (ARB), both CCB and ARB, both CCB and beta blocker, both ARB and beta blocker, and other drugs, respectively. In general, randomization was well done between the two groups. Age; height; gender; weight; body mass index; systolic and diastolic BP; urine excretion; urinary Na, K, and creatinine excretions; and urinary Na/K ratio were not significantly different between the two groups (Table 1). At baseline, 24-hour urinary Na/K ratios were 3.78 and 3.64 in the intervention group and control group, respectively; corresponding values were 198.6 mmol/24 hours and 191.4 mmol/24 hours for Na excretion, 56.6 mmol/24 hours and 53.7 mmol/24 hours for K excretion, 125.9 mm Hg and 125.8 mm Hg for systolic BP, 79.3 mm Hg and 77.9 mm Hg for diastolic BP, and 23.2 and 23.5 for body mass index (Table 1 and Table 2).

**Table 1.** Baseline characteristics of study participants, 2012

Factors	Intervention group	Control group	*P* value
	(*n* = 46)	(*n* = 46)	
	
	Mean (SE)	Mean (SE)	
Age	55.0 (1.2)	54.0 (1.2)	NS
Height, cm	164.4 (1.3)	162.7 (0.9)	NS
Male, %	47.8	47.8	NS
Weight, kg	63.0 (1.8)	62.5 (1.7)	NS
BMI, kg/m^2^	23.2 (0.5)	23.5 (0.5)	NS
Urine excretion, mL/24 hours	1,838 (97)	1,784 (127)	NS
Urinary Na/K ratio, mmol/mmol	3.78 (0.23)	3.64 (0.15)	NS
Urinary Na excretion, mmol/24 hours	198.6 (11.8)	191.4 (11.4)	NS
Urinary K excretion, mmol/24 hours	56.6 (3.3)	53.7 (2.5)	NS
Urinary creatinine excretion, mg/24 hours	1,458 (58)	1,407 (55)	NS
Systolic blood pressure, mm Hg	125.9 (2.4)	125.8 (2.1)	NS
Diastolic blood pressure, mm Hg	79.3 (1.7)	77.9 (1.3)	NS
Hypertensive patients, %	23.9	19.6	NS
Anti-hypertensive medication, %	15.2	17.4	NS

BMI, body mass index; Na, sodium; K, potassium; NS, not statistically significant; SE, standard error.

**Table 2.** Intervention outcomes at baseline and 1-month outcome assessments

Intervention Outcomes		Intervention group	Control group	Difference between two sub groups (intervention-control)	
		(*n* = 46)	(*n* = 46)		
		
		Mean (SD)	Mean (SD)	Mean (95% CI)	*P* value
Urinary Na/K molar ratio	Before Intervention	3.78 (1.59)	3.64 (1.04)		
	After Intervention	3.22 (1.34)	3.59 (1.27)		
	Change	−0.55 (1.50)	−0.06 (1.25)	−0.50 (−1.07, 0.08)	0.088

Urinary Na excretion, mmol/24 hours	Before Intervention	198.6 (79.7)	191.4 (77.6)		
	After Intervention	180.1 (77.6)	182.7 (74.7)		
	Change	−18.5 (63.6)	−8.7 (82.2)	−9.7 (−40.1, 20.7)	0.528

Urinary K excretion, mmol/24 hours	Before Intervention	56.6 (22.6)	53.7 (17.1)		
	After Intervention	59.2 (21.0)	52.2 (17.2)		
	Change	2.6 (19.5)	−1.5 (17.4)	4.0 (−3.6, 11.7)	0.300

Systolic blood pressure, mm Hg	Before Intervention	125.9 (17.1)	125.8 (15.5)		
	After Intervention	122.5 (17.6)	123.6 (14.8)		
	Change	−3.4 (9.7)	−2.2 (8.5)	−1.3 (−5.0, 2.5)	0.512

Diastolic blood pressure, mm Hg	Before Intervention	79.3 (12.2)	77.9 (9.6)		
	After Intervention	77.6 (12.0)	76.9 (9.6)		
	Change	−1.7 (6.0)	−1.0 (4.2)	−0.7 (−2.9, 1.4)	0.505

Weight, kg	Before Intervention	62.8 (12.1)	62.3 (11.2)		
	After Intervention	62.9 (12.2)	62.2 (11.3)		
	Change	0.0 (0.9)	−0.2 (1.0)	0.2 (−0.2, 0.5)	0.394

CI, confidence interval; Na, sodium; K, potassium; SD, standard deviation.

### Effects on primary outcome: change in urinary Na/K ratio

No participant dropped out during the 1-month intervention period. Mean measurement frequency for monitor use during the intervention was 2.8 times/day. After the intervention period, there was a reduction in the urinary Na/K ratio of 0.55 (SD 1.50) in the intervention group and 0.06 (SD 1.25) in the control group (*P* = 0.088, Table 2).

### Effects on secondary outcomes

Na excretion reduction was 18.5 mmol/24-hours (1.1 g of salt per day) and 8.7 mmol/24-hours (0.5 g of salt per day) (*P* = 0.528) in the intervention group and the control group, respectively, while K excretion increased by 2.6 mmol/24-hours in the intervention group and decreased by 1.5 mmol/24-hours in the control group (*P* = 0.30) (Table 2). There were no significant reductions observed in either BP or body weight after the intervention (Table 2). There was no significant difference in changes in systolic (*P* = 0.512) or diastolic BP (*P* = 0.505) or body weight (*P* = 0.394) between the intervention group and the control group (Table 2). No serious adverse events were associated with the intervention.

## DISCUSSION

This was the first study to report on the effectiveness of using a monitoring device for casual urinary Na/K ratio over a 1-month intervention for the purpose of reducing Na and increasing K through self-monitoring. Prior to this report, effect of self-monitoring and the association with dietary improvement remained unknown. We recruited healthy and hypertensive individuals in this study, since reducing Na/K ratio is essential for preventing hypertension and CVDs prior to clinical onset.^5^^–^^18^^,^^37^ The main findings of this first study on self-monitoring Na/K ratio among healthy and hypertensive individuals included a trend towards larger reductions in urinary Na/K ratio observed in the intervention group. However, the effect of the self-monitoring device on the urinary Na/K ratio did not reach the accepted level of statistical significance on the pre-specified effect size (target reduction of 1 unit difference of Na/K ratio between intervention and control groups) under “pure self-management” (ie, without additional professional support) (*P* = 0.088). There were no significant reductions observed for Na and K excretions, BP, or body weight.

In earlier studies, researchers attempted to use dietary intervention as a way of evaluating the effects of an increased fruit and vegetable intake, while other groups used special diets, such as the DASH-sodium diet, in order to prevent non-communicable diseases.^38^^–^^41^ Since most of these previous interventions showed positive effects,^38^^–^^41^ international recommendations have advised increasing fruit and vegetable intake.^42^^,^^43^ Although dietary interventions with larger effects tended to depend on the intensity of the intervention, some of these intensive interventions can be very difficult and expensive to apply in outpatient practice.^39^^,^^44^ As a way of balancing a lower effort and financial burden while maintaining an effective intervention, “pure self-management” using a self-monitoring device is one of the methods that could be used to help improve dietary habits. Positive effects have been demonstrated when using this technique in BP and glucose monitoring.^45^^–^^47^ Unfortunately, there are few tools that are capable of providing an easy and accurate measurement that can be used for self-monitoring the dietary habit. In this study, we used a newly developed urinary Na/K ratio monitor providing onsite accurate feedback within a minute that can be used to evaluate the intervention effect of “pure self-management” by healthy and hypertensive subjects.^30^

In this present study, there was a larger reduction of the Na/K ratio for the “device + usual care” intervention group versus the “usual care” control group. However, the intervention effect for our participants was half of what we originally expected; the initial sample size was subsequently insufficient, which led to deficiency of power for detecting statistical significance towards the pre-specified target reduction on urinary Na/K ratio having a 1 unit difference in Na/K ratio between intervention and control groups. The baseline value of urinary Na excretion was close to our assumption; however, the urinary Na/K ratio was lower and the urinary K excretion was higher than initially expected. Since we are the first to report on the effectiveness of using a monitoring device for casual urinary Na/K ratio and could not find any previous intervention studies reporting dietetic education on Na/K ratio, it was difficult to estimate the baseline values and the effect size of the intervention precisely and calculate an appropriate sample size in this preliminary stage trial. We minimally referred to findings from observational studies to hypothesize the intervention effect in this study; however, the pre-specified target reduction in Na/K ratio set with our expectations might have been overambitious to achieve among free-living healthy and hypertensive Japanese individuals. “Pure self-management” works with individual’s knowledge, skills, and motivation toward behavior change; however, it seemed difficult for Japanese study participants to achieve the level of Na/K ratio observed in Western countries. This discrepancy may be due to difficulties with food choice in local grocery stores, limited amount of vegetables and fruits available during the winter period, or because avoiding high-salt content foods might have been challenging with only a brief education. Since self-monitoring devices are different from therapeutic devices, urinary Na/K ratio monitor may be powerless without practical dietary programs which support individuals to acquire appropriate skills and practice lowering Na/K ratio effectively. Hence, the present null findings do not necessarily suggest that self-monitoring of the Na/K ratio is not an effective method for dietetic intervention. Recruiting participants from the general population, who have no special skill or experience using the device or performing “pure self-management”, may be one of the limitations of this study that had led to the observed weaker intervention effect. Thus, we cannot deny the possibility of reducing Na/K ratio using a self-monitoring device based on the present null findings. However, effective methods to enlarge the synergetic effect of dietary program and self-monitoring practices would be essential for lowering individuals’ Na/K ratios. Further research is needed to improve the intervention effect to achieve the reduction. If we recalculate the sample size based on actual effect size (target reduction of 0.5 unit difference of Na/K ratio between intervention and control groups), at least 147 participants in each group would have been needed to detect a significant difference between the intervention and control groups. However, the current results suggest that self-monitoring could potentially reduce the Na/K ratio with relatively little burden, as it can be easily implemented in normative daily practice.

Focusing on differences between the present study and previously reported dietary interventions may provide important clues on how to enhance the intervention effect.^39^ Introducing active advice from a nutritionist with appropriate timing may help individuals to better understand how they can properly improve their dietary habits.^44^ In this context, acquiring basic knowledge and skills for Na reduction and K increase might be one of the key factors to improve individuals’ confidence and enhance their motivation toward dietary improvements. Thus, it is reasonable to infer that, in combination with the conventional population approach, the individual approach (such as self-monitoring of the urinary Na/K ratio at home, brief expertise consultancy, and/or remote educational feedback systems providing useful feedback to individuals) may support the timely achievement of goals, such as that set by WHO of less than 2 g of Na per day by 2025.^24^

One potential limitation of this study is that participants were free-living middle-aged men and women. As such, the majority were not subjected to a diet with continuous salt reduction or a controlled diet. Hence, it is not known whether these findings are also applicable to the elderly and patients under continuous salt reduction practice.

In conclusion, the intervention effect of self-monitoring urinary Na/K ratio under “pure self-management” settings resulted in half of the pre-specified effect size between intervention and control groups and did not achieve the usually accepted level of statistical significance towards the pre-specified targeted reduction of the ratio (1 unit difference of Na/K ratio) in this first randomized trial. The pre-specified effect size for reducing Na/K ratio might have been overambitious rather than realistic; however, there might be a possibility showing no clinically important effect of self-monitoring urinary Na/K ratio under “pure self-management” settings. Further study is needed to explore an effective method to enhance the synergetic effect of dietary education program and self-monitoring practice to achieve reduction of the Na/K ratio.

## APPENDIX A. SUPPLEMENTARY DATA

eFigure 1. Flow diagram of study participants in this study, satisfying the elements required in CONSORT 2010 statements which relate to eTable 1.

eFigure 2. Pearson’s correlation coefficient between the urinary Na/K ratio measured by the portable handy-sized urinary Na/K ratio monitor (HEU-001F, OMRON Healthcare Co., Ltd., Kyoto, Japan) and the biochemical inspection center (BML Inc., Tokyo, Japan).

eTable 1. Checklist for the elements required in CONSORT 2010 statements, and the corresponding statements written in this manuscript
