# Supplementary figures and images for: Effectiveness of a Self-monitoring Device for Urinary Sodium-to-Potassium Ratio on Dietary Improvement in Free-Living Adults: a Randomized Controlled Trial
Source: J Epidemiol. 2018 Jan 5;28(1):41–7. doi: 10.2188/jea.JE20160144 (PMC5742378; doi:10.2188/jea.JE20160144)

**eFigure 1.**

**CONSORT 2010 Flow Diagram**

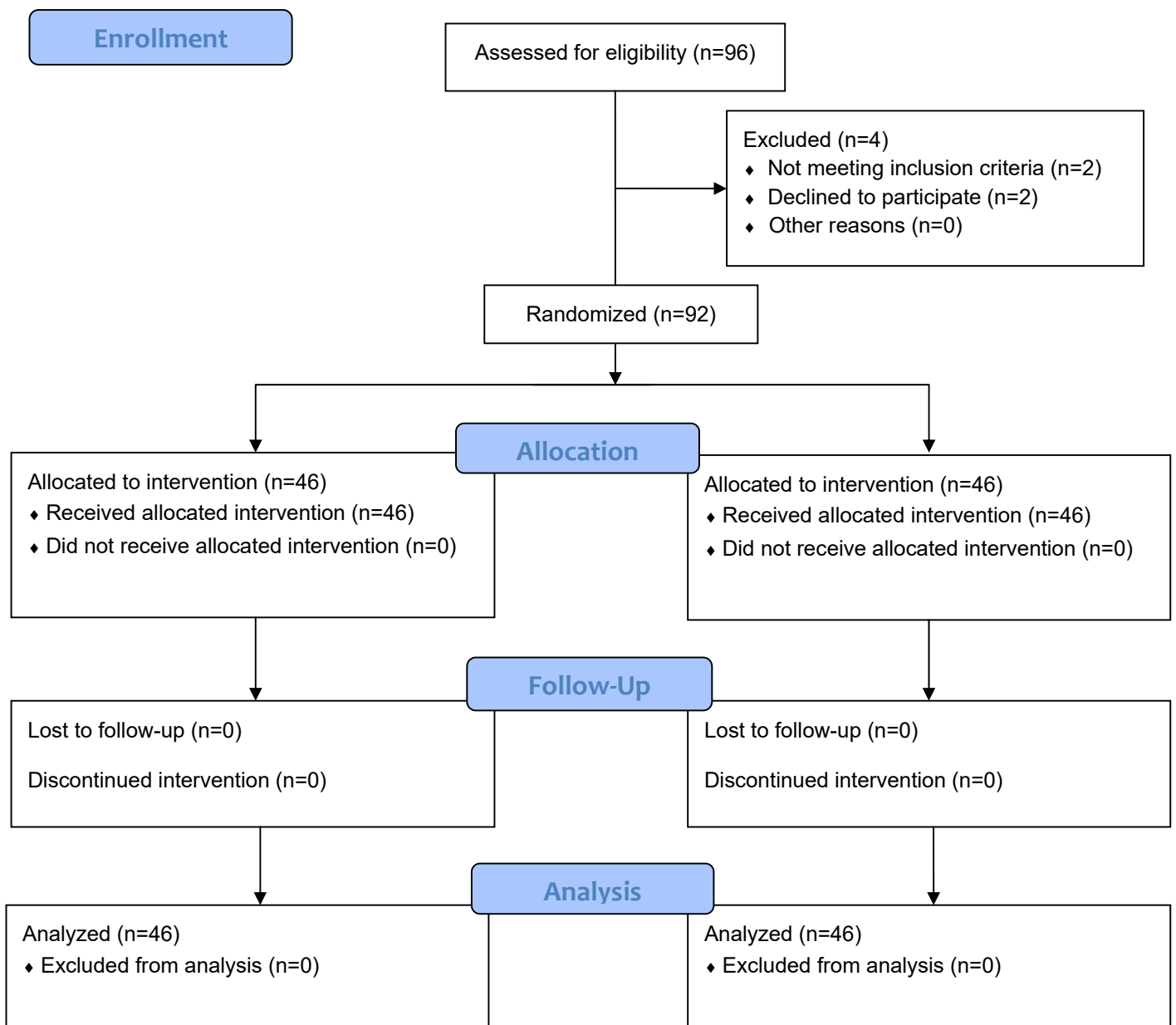

eFigure 2.

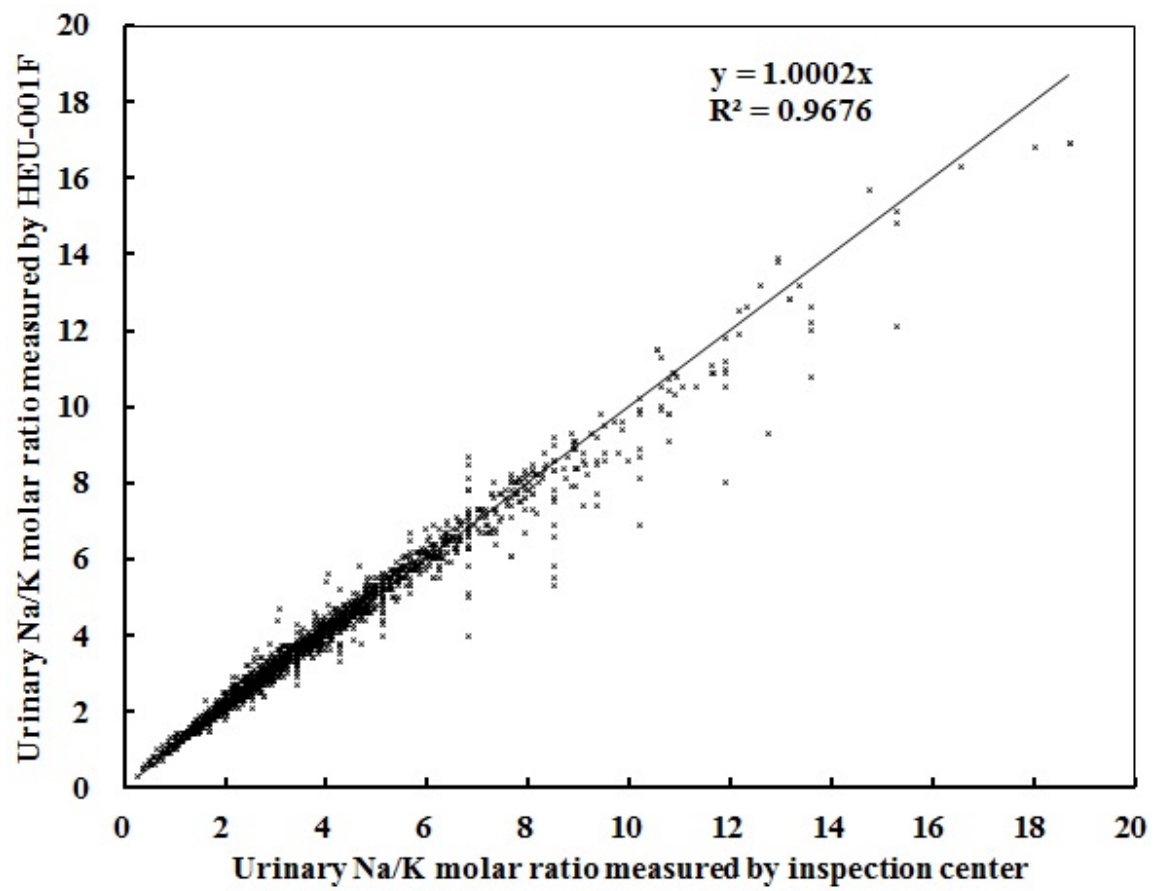

Supplement: Supplementary file 1 [file je-28-041-s001.pdf]
